# Impact of brain‐derived neurotrophic factor genetic polymorphism on cognition: A systematic review

**DOI:** 10.1002/brb3.1009

**Published:** 2018-06-01

**Authors:** Yi Long Toh, Terence Ng, Megan Tan, Azrina Tan, Alexandre Chan

**Affiliations:** ^1^ Department of Pharmacy Faculty of Science National University of Singapore Singapore Singapore; ^2^ Department of Pharmacy National Cancer Centre Singapore Singapore Singapore

**Keywords:** attention, brain‐derived neurotrophic factor, executive control, memory, neuroprotection

## Abstract

**Introduction:**

Brain‐derived neurotrophic factor (BDNF) has an important role in the neurogenesis and neuroplasticity of the brain. This systematic review was designed to examine the association between BDNF Val66Met (rs6265) polymorphism and four cognitive domains—attention and concentration, executive function, verbal fluency, and memory, respectively.

**Methodology:**

Primary literature search was performed using search engines such as PubMed and Scopus. Observational studies that evaluated the neurocognitive performances in relation to BDNF polymorphism within human subjects were included in this review, while animal studies, overlapping studies, and meta‐analysis were excluded.

**Results:**

Forty of 82 reviewed studies (48.8%) reported an association between Val66Met polymorphism and neurocognitive domains. The proportion of the studies showing positive findings in cognitive performances between Val/Val homozygotes and Met carriers was comparable, at 30.5% and 18.3%, respectively. The highest percentage of positive association between Val66Met polymorphism and neurocognition was reported under the memory domain, with 26 of 63 studies (41.3%), followed by 18 of 47 studies (38.3%) under the executive function domain and four of 23 studies (17.4%) under the attention and concentration domain. There were no studies showing an association between Val66Met polymorphism and verbal fluency. In particular, Val/Val homozygotes performed better in tasks related to the memory domain, while Met carriers performed better in terms of executive function, in both healthy individuals and clinical populations.

**Conclusion:**

While numerous studies report an association between Val66Met polymorphism and neurocognitive changes in executive function and memory domains, the effect of Met allele has not been clearly established.

## 1 INTRODUCTION

The brain‐derived neurotrophic factor (BDNF) belongs to the neurotrophin superfamily and has an essential role in the neurogenesis and neuroplasticity of the brain (Matsuo et al., 2009). The signaling BDNF supports the survival of existing neurons and encourages the proliferation and differentiation of new neurons and synapses in both the central and peripheral nervous system (Acheson et al., 1995; Huang & Reichardt, 2001). In the brain, BDNF is highly expressed in the hippocampus, cortex, and basal forebrain and has an important role in regions that are vital to learning and memory (Bekinschtein, Cammarota, & Katche, 2008).

The expression of BDNF is encoded by the BDNF gene which has numerous polymorphisms. BDNF gene encodes for the precursor polypeptide (pre‐pro‐BDNF) which is proteolytically cleaved to generate pro‐BDNF and mature BDNF. Pro‐BDNF is known to modulate neuronal structure and long‐term hippocampal‐plasticity (Bath & Lee, 2006; Binder & Scharfman, 2004). The most extensively studied single nucleotide polymorphism (SNP) in the BDNF gene is the G196A polymorphism (rs6265). The G196A polymorphism occurs in the proregion of BDNF, resulting in an amino acid substitution of valine for methionine at codon position 66 (Val66Met). This polymorphism change has been linked to aberrant sorting of pro‐BDNF into secretory vesicles and decreased activity‐dependent secretion of BDNF which constitutes the main process in the regulation of extracellular levels of BDNF (Wei et al., 2012). There has also been suggestion by a study that this polymorphism may influence the interaction between mature and pro‐BDNF, affecting the stability of the complex formed between the two and attenuating the ability of BDNF to inhibit hippocampal long‐term depression (Uegaki et al., 2017).

In view of the importance of BDNF in the central nervous system (CNS) and the functional consequences of its SNP, numerous genetic studies have evaluated the association between the BDNF rs6265 polymorphism with cognitive performance in various disease states. Recent studies have also focused their interest in implications of BDNF on the pathogenesis of brain‐related disorders such as neurodegenerative and psychiatric diseases (Adachi, Numakawa, Richards, Nakajima, & Kunugi, 2014; Yang, Ren, Zhang, Chen, & Hashimoto, 2017). As BDNF is highly expressed in regions of the CNS that modulate learning and memory, it would be of interest to understand how the BDNF rs6265 polymorphism influence specific cognitive domains such as the attention and concentration, executive function, verbal fluency, and memory.

Therefore, this systematic review was conducted to evaluate the relevant studies regarding associations between the SNP Val66Met and neurocognitive domains.

## 2 METHODS

### 2.1 Literature search strategy

A systematic primary literature search was conducted using PubMed and Scopus in July 2015. The search included articles published exclusively in English dated up to 1 May 2017. No screening limits were imposed in terms of the publication dates. The literature search was performed using Medical Subject Headings and from combinations of relevant keywords, such as “BDNF,” “brain‐derived neurotrophic factor,” “Val66Met,” “polymorphism,” and “neurocognition”. Bibliographies of relevant articles were also reviewed.

### 2.2 Inclusion/exclusion criteria

Published studies were included into the systematic review if they fulfilled the following inclusion criteria: (a) evaluated cognitive performance in relation to the BDNF rs6265 polymorphism in either a healthy population or a specific clinical population, (b) reported the allele and genotype distribution in the study population, and (c) utilized traditional neuropsychological batteries to assess cognitive function.

In vitro research, animal research, editorials, systematic reviews, and meta‐analysis were excluded. Studies were also excluded if the Mini Mental State Examination (MMSE) and Intelligence Quotient (IQ) have been used to evaluate cognitive performance in the human population. This is because MMSE not only lacks sensitivity in identifying mild cognitive impairment, but the scores are also influenced by demographic factors such as age, education, cultural, and socioeconomic background. Conversely, studies utilizing IQ test as an outcome were excluded because IQ test is not a comprehensive method for evaluating the various cognitive domains. Studies that utilized functional magnetic resonance imaging (fMRI) as the sole outcome to measure brain functioning were also excluded.

### 2.3 Data extraction

Three investigators independently extracted the data using a predesigned, piloted form. Each study was reviewed, and the relevant information was extracted and compiled. The following information was extracted from each study: study objectives, study design, study population characteristics (disease states, age, gender, and education level), total number of study participants, genotype distribution, and the type of outcome measures.

The review was carried out to evaluate the effect of Val66Met polymorphism on four cognitive domains: (a) attention and concentration, (b) executive function, (c) verbal fluency, and (d) memory.

The attention and concentration domain determine the content of consciousness and influence the quality of conscious experience, while the executive function domain represents the ability to reason, plan, and execute. Verbal fluency domain is related to verbal functioning, which involves the semantic and phonemic fluency. Memory encompasses a broad domain which includes declarative memory tasks for verbal tasks, associative memory as well as working memory that can be used for arithmetical processing.

Due to the huge heterogeneity in terms of study design, study population, and the outcome measurements among the various reviewed studies, meta‐analysis was not performed in this review.

## 3 RESULTS

**Figure 1:**
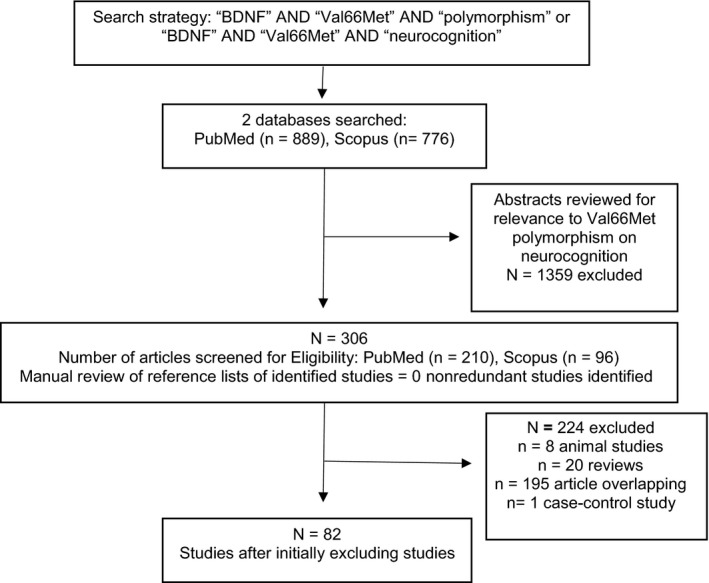
Flow chart of the inclusion and exclusion process of the study

A total of 1,665 articles were identified. Irrelevant studies were excluded through abstracts review and evaluation of the nature of the studies. A total of 82 unique articles fulfilled the predetermined inclusion–exclusion criteria (Figure 1). The number of studies evaluating each cognitive domain was identified; attention and concentration (*n* = 23), executive function (*n* = 47), verbal fluency (*n* = 18), and memory (*n* = 63). The included studies examined genetic association with neurocognition in healthy patients and other disease states. In total, 40 of the 82 reviewed studies (48.8%) (Aas et al., 2013; Alfimova, Korovaitseva, Lezheiko, & Golimbet, 2012; Altmann et al., 2016; Avgan et al., 2017; Barbey et al., 2014; Canivet et al., 2015; Cao et al., 2016; De Beaumont, Fiocco, Quesnel, Lupien, & Poirier, 2013; Egan, Kojima, & Callicott, 2003; Freundlieb et al., 2015; Gajewski, Hengstler, Golka, Falkenstein, & Beste, 2011; Gong et al., 2009; Gonzalez et al., 2016; Gonzalez‐Giraldo et al., 2014; Gosselin et al., 2016; Ho, Andreasen, Dawson, & Wassink, 2007; Huang et al., 2014; Jasinska et al., 2016; Kim et al., 2016; Lamb, Thompson, McKay, Waldie, & Kirk, 2015; Lee, Baek, & Kim, 2015; Lim et al., 2013, 2016; Miyajima et al., 2008; McAllister et al., 2012; Nagata, Shinagawa, Nukariya, Yamada, & Nakayama, 2012; Narayanan et al., 2016; Ng et al., 2016; Raz, Rodrigue, Kennedy, & Land, 2009; Richter‐Schmidinger et al., 2011; Rybakowski, Borkoswka, Czerski, Skibinska, & Hauser, 2003; Rybakowski et al., 2006; Schofield et al., 2009; Szabo et al., 2013; Tan et al., 2005; Tukel et al., 2012; van der Kolk et al., 2015; Yin, Hou, Wang, Sui, & Yuan, 2015; Yogeetha et al., 2013; Zhang et al., 2012) reported a significant association between Val66Met polymorphism and the neurocognitive domains of interest.

### 3.1 Attention/concentration domain

A total of 23 studies evaluated the association between the BDNF polymorphism and the attention/concentration domain (Alfimova et al., 2012; Cherubin et al., 2014; da Rocha, Malloy‐Diniz, Lage, & Correa, 2011; Dennis et al., 2011; Freundlieb et al., 2012; Freundlieb et al., 2015; Gatt et al., 2007; Ho et al., 2006; Huang et al., 2014; Kim et al., 2016; Lim et al., 2013; Lin et al., 2016; Mezquida et al., 2016; Narayanan et al., 2016; Oroszi et al., 2006; Schofield et al., 2009; Swardfager et al., 2011; Szabo et al., 2013; Thow, Summers, Summers, Saunders, & Vickers, 2017; Tukel et al., 2012; Ward et al., 2015; Yu et al., 2008; Zhang et al., 2012). Ten of these studies focused on healthy subjects (Alfimova et al., 2012; Cherubin et al., 2014; Dennis et al., 2011; Freundlieb et al., 2012, 2015; Gatt et al., 2007; Huang et al., 2014; Lim et al., 2013; Schofield et al., 2009; Ward et al., 2015), and the rest evaluated samples of different disease states such as Alzheimer’s disease (*n* = 1) (Lin et al., 2016), cardiovascular diseases (*n* = 2) (Swardfager et al., 2011; Szabo et al., 2013), obsessive‐compulsive disorder (*n* = 2) (da Rocha et al., 2011; Tukel et al., 2012), schizophrenia (*n* = 5) (Ho et al., 2006; Kim et al., 2016; Mezquida et al., 2016; Zhang et al., 2012, 2016), traumatic brain injury (*n* = 2) (Narayanan et al., 2016; Yu et al., 2008), and systemic lupus erythematous (*n* = 1) (Oroszi et al., 2006).

Among the reviewed studies, four studies (17.4%) (Kim et al., 2016; Narayanan et al., 2016; Szabo et al., 2013; Zhang et al., 2012) observed significant positive association between BDNF Val66Met polymorphism and the attention/concentration domain. In a study conducted in patients with mild traumatic brain injury, Val/Val homozygotes were reported to perform better in domains of attention/concentration over time (Narayanan et al., 2016). Conversely, Met carriers were reported to perform better than Val/Val homozygotes in attention/concentration domain in another study focusing on elderly population with cardiovascular diseases (Szabo et al., 2013). In studies conducted in the schizophrenic population, one reported that Val/Val homozygotes had better attention performance than carriers of Met/Met homozygotes (Zhang et al., 2012), while another study reported the association of Val allele with poorer neurocognitive function in the attention/concentration domain (Kim et al., 2016).

### 3.2 Executive function domain

Forty‐seven relevant studies were identified within the executive function domain. Twenty studies evaluated on healthy subjects (Alfimova et al., 2012; Cherubin et al., 2014; Dennis et al., 2011; De Beaumont et al., 2013; Erickson et al., 2013; Freundlieb et al., 2012; Freundlieb et al., 2015; Gong et al., 2009; Gajewski et al., 2011; Ghisletta et al., 2014; Gonzalez et al., 2016; Harris et al., 2006; Huang et al., 2014; Lim et al., 2013; Schofield et al., 2009; Thibeau, McFall, Wiebe, Anstey, & Dixon, 2016; Thow et al., 2017; Ward et al., 2014, 2015; Wilkosc et al., 2016), and the rest evaluated samples of different disease states such as Alzheimer’s disease (*n* = 4) (Lee et al., 2015; Lin et al., 2016; Nagata et al., 2011, 2012), Parkinson’s disease (*n* = 3) (Altmann et al., 2016; Bialecka et al., 2014; van der Kolk et al., 2015), cardiovascular diseases (*n* = 2) (Swardfager et al., 2011; Szabo et al., 2013), obsessive‐compulsive disorder (*n* = 2) (da Rocha et al., 2011; Tukel et al., 2012), schizophrenia and bipolar disorder (*n* = 7) (Egan et al., 2003; Ho et al., 2006; Kim et al., 2016; Lee et al., 2016; Mezquida et al., 2016; Rybakowski et al., 2003, 2006), traumatic brain injury (*n* = 4) (Barbey et al., 2014; McAllister et al., 2012; Narayanan et al., 2016; Yu et al., 2008), depression (*n* = 1) (Yin et al., 2015), multiple sclerosis (*n* = 1) (Fera et al., 2013), breast cancer patients (*n* = 1) (Ng et al., 2016), systemic lupus erythematosus (*n* = 1) (Oroszi et al., 2006), and psychosis (*n* = 1) (Aas et al., 2013).

Out of the 47 studies, 18 studies (38.3%) (Alfimova et al., 2012; Altmann et al., 2016; Barbey et al., 2014; Freundlieb et al., 2015; Gonzalez et al., 2016; Kim et al., 2016; Lim et al., 2013; Lee et al., 2015; McAllister et al., 2012; Nagata et al., 2012; Narayanan et al., 2016; Ng et al., 2016; Rybakowski et al., 2003, 2006; Szabo et al., 2013; Tukel et al., 2012; van der Kolk et al., 2015; Yin et al., 2015) reported significant association between BDNF Val66Met polymorphism and the executive function domain. Among these studies, 11 studies (23.4%) (Alfimova et al., 2012; Barbey et al., 2014; Freundlieb et al., 2015; Gonzalez et al., 2016; Kim et al., 2016; van der Kolk et al., 2015; McAllister et al., 2012; Nagata et al., 2012; Ng et al., 2016; Szabo et al., 2013; Yin et al., 2015) reported that Met carriers performed significantly better than Val/Val homozygotes in healthy controls and populations with Alzheimer’s disease, Parkinson’s disease, cardiovascular diseases, traumatic brain injury, and depression. One study reported that genotypes of Met/Met homozygotes performed the worst in executive functions as compared to Val/Met genotypes and Val/Val genotypes (McAllister et al., 2012). In contrast, seven studies (14.9%) (Altmann et al., 2016; Lee et al., 2015; Lim et al., 2013; Narayanan et al., 2016; Rybakowski et al., 2003, 2006; Tukel et al., 2012) showed that genotypes of Val/Val homozygotes have significantly better executive function than Met carriers in healthy adults and populations with Parkinson’s disease, traumatic brain injury, obsessive‐compulsive disorder, and bipolar disorder.

### 3.3 Verbal fluency domain

Eighteen studies were identified when evaluating BDNF Val66Met polymorphism within the verbal fluency domain. These studies evaluated populations of different disease states such as healthy controls (*n* = 11) (Alfimova et al., 2012; De Beaumont et al., 2013; Gajewski et al., 2011; Gong et al., 2009; Freundlieb et al., 2012, 2015; Harris et al., 2006; Huang et al., 2014; Karnik, Wang, Barch, Morris, & Csernansky, 2010; Schofield et al., 2009; Ward et al., 2015), traumatic brain injury (*n* = 1) (Yu et al., 2008), obsessive‐compulsive disorder (*n* = 1) (Tukel et al., 2012), psychosis (*n* = 2) (Aas et al., 2013; Martinho et al., 2012), Parkinson’s disease (*n* = 1) (van der Kolk et al., 2015), cardiovascular diseases (*n* = 1) (Szabo et al., 2013), and breast cancer patients (*n* = 1) (Ng et al., 2016). In all 18 of the identified studies, none of the studies observed an association between the BDNF Val66Met polymorphism and verbal fluency domain.

### 3.4 Memory domain

Sixty‐three studies were identified under the memory domain. The included studies had healthy controls (*n* = 36) (Alfimova et al., 2012; Avgan et al., 2017; Beste, Schneider, Epplen, & Arning, 2011; Bombardier, Beauchemin, Gosselin, Poirier, & De Beaumont, 2016; Cathomas, Vogler, Euler‐Sigmund, de Quervain, & Papassotiropoulos, 2010; Canivet et al., 2015; Chen et al., 2015, 2016; Dennis et al., 2011; De Beaumont et al., 2013; Erickson et al., 2013; Freundlieb et al., 2015; Gajewski et al., 2011; Gong et al., 2009; Gonzalez‐Giraldo et al., 2014; Gosselin et al., 2016; Huang et al., 2014; Jasinska et al., 2016; Karnik et al., 2010; Kennedy et al., 2015; Lim et al., 2013; Liu et al., 2014; Lamb et al., 2015; Miyajima et al., 2008; Montag et al., 2014; Raz et al., 2009; Richter‐Schmidinger et al., 2011; Schofield et al., 2009; Stuart, Summers, Valenzuela, & Vickers, 2014; Thow et al., 2017; Voineskos et al., 2011; Ward et al., 2014, 2015; Wilkosc et al., 2016; Wegman, Tyborowska, Hoogman, Arias Vásquez, & Janzen, 2016; Yogeetha et al., 2013), dementia (*n* = 1) (Kim et al., 2015), Alzheimer’s (*n* = 3) (Gomar, Conejero‐Goldberg, Huey, Davies, & Goldberg, 2016; Lim et al., 2016; Lin et al., 2016), Parkinson’s (*n* = 1) (Bialecka et al., 2014), cardiovascular disease (*n* = 2) (Swardfager et al., 2011; Szabo et al., 2013), obsessive‐compulsive disease (*n* = 1) (Tukel et al., 2012), multiple sclerosis (*n* = 1) (Fera et al., 2013), psychosis (*n* = 2) (Aas et al., 2013; Martinho et al., 2012), depression/anxiety disorders (*n* = 2) (Molendijk et al., 2012; Strauss, Barr, & George, 2004), brain injury (*n* = 2) (McAllister et al., 2012; Narayanan et al., 2016), amnestic mild cognitive impairment (*n* = 1) (Yu et al., 2008), schizophrenia and bipolar disorders (*n* = 9) (Cao et al., 2016; Egan et al., 2003; Ho et al., 2006, 2007; Kim et al., 2016; Mezquida et al., 2016; Rybakowski et al., 2003; Tan et al., 2005; Zhang et al., 2016), systematic lupus erythematosus (*n* = 1) (Oroszi et al., 2006), as well as patients receiving treatment of gonadotrophin‐releasing hormone (*n* = 1) (Wei et al., 2017). Among the reviewed studies, 26 studies (41.3%) (Alfimova et al., 2012; Aas et al., 2013; Avgan et al., 2017; Canivet et al., 2015; Cao et al., 2016; De Beaumont et al., 2013; Egan et al., 2003; Gajewski et al., 2011; Gong et al., 2009; Gonzalez‐Giraldo et al., 2014; Gosselin et al., 2016; Ho et al., 2007; Huang et al., 2014; Jasinska et al., 2016; Kim et al., 2016; Lamb et al., 2015; Lim et al., 2016; Miyajima et al., 2008; Narayanan et al., 2016; Raz et al., 2009; Richter‐Schmidinger et al., 2011; Schofield et al., 2009; Szabo et al., 2013; Tukel et al., 2012; Tan et al., 2005; Yogeetha et al., 2013) observed significant positive association between BDNF Val66Met polymorphism and memory domain.

Nineteen of these identified studies (30.2%) (Aas et al., 2013; Canivet et al., 2015; Cao et al., 2016; De Beaumont et al., 2013; Egan et al., 2003; Gong et al., 2009; Gosselin et al., 2016; Ho et al., 2006; Jasinska et al., 2016; Lamb et al., 2015; Lim et al., 2016; Miyajima et al., 2008; Narayanan et al., 2016; Richter‐Schmidinger et al., 2011; Raz et al., 2009; Schofield et al., 2009; Tan et al., 2005; Tukel et al., 2012; Yogeetha et al., 2013) favored Val/Val homozygotes under the memory domain, in healthy controls and populations with psychosis, depression, Alzheimer’s disease, brain injury, obsessive‐compulsive disorder, and schizophrenia, respectively. Conversely, Met carriers were found to perform better in memory tasks in seven of such studies (11.1%) that evaluate healthy controls and populations with cardiovascular diseases and schizophrenia (Alfimova et al., 2012; Avgan et al., 2017; Gajewski et al., 2011; Gonzalez‐Giraldo et al., 2014; Huang et al., 2014; Kim et al., 2016; Szabo et al., 2013).

## 4 DISCUSSION

To our best knowledge, this is the first systematic review conducted to evaluate the association between Val66Met polymorphism and various cognitive domains. Approximately half of the reviewed studies indicated a presence of association with cognitive domains in comparison with a meta‐analysis which also did not manage to establish significant genetic associations between Val66Met polymorphism with cognitive domains of general cognitive ability, memory, executive functioning, and visual and cognitive fluency (Mandelman & Grigorenko, 2012). Across all four domains that were studied, positive studies do not appear to favor either the Val/Val homozygote genotype or Met carriers because the proportions of studies showing an association are comparable, with 25 studies (30.5%)^1^ Val/Val homozygote genotype and 15 studies (18.3%)^1^ favoring that of Met carriers (Table 1). Without an association being clearly established, it remains unclear as to whether the Val66Met polymorphism would confer a neuroprotective effect. However, the higher proportion of reviewed studies reporting an association suggests that BDNF polymorphism has an influence on neurocognition.

**Table 1:** Summary of literature that evaluated the association between Val66Met polymorphism and the respective neurocognitive domains for (a) Overall, (b) Healthy individuals and (c) Clinical populations

(a)				
Domains of interest	Attention and concentration (%)	Executive function (%)	Verbal fluency	Memory (%)
Total studies	23	47	18	63
Presence of association	4 (17.4)	18 (38.3)	0	26 (41.3)
Extent of association				
Favoring Val/Val homozygotes	2 (8.70)	7 (14.9)	0	19 (30.2)
Favoring Met carriers (Val/Met and Met/Met)	2 (8.70)	11 (23.4)	0	7 (11.1)

(b)				
Domains of interest (*n* = total)	Attention and concentration (*n* = 23)	Executive function (*n* = 47) (%)	Verbal fluency (*n* = 18)	Memory (*n* = 63) (%)
Number of studies	10	20	11	36
Presence of association	0	4 (8.51)	0	16 (25.4)
Extent of association				
Favoring Val/Val homozygotes	0	1 (2.12)	0	11 (17.5)
Favoring Met carriers (Val/Met and Met/Met)	0	3 (6.40)	0	5 (7.94)

(c)				
Domains of interest (*n*=total)	Attention and concentration (*n* = 23) (%)	Executive function (*n* = 47) (%)	Verbal fluency (*n* = 18)	Memory (*n* = 63) (%)
Number of studies	13	27	7	27
Presence of association	4 (17.4)	14 (29.8)	0	10 (15.9)
Extent of association				
Favoring Val/Val homozygotes	2 (8.70)	6 (12.8)	0	8 (12.7)
Favoring Met carriers (Val/Met and Met/Met)	2 (8.70)	8 (17.0)	0	2 (3.17)

Among the various cognitive domains evaluated, the domain of memory has the strongest evidence to suggest the role of the Val66Met polymorphism in neurocognition. Overall, 19 of the reviewed studies reported Val/Val homozygotes performing better in memory‐related tasks compared to Met carriers who were favored in seven of the total studies. This trend of higher proportion of studies favoring Val/Val homozygotes was also consistent in both studies of healthy individuals (Table 2) and clinical populations (Table 3). The findings that Met carriers are more likely to be adversely affected in terms of memory are aligned with the results from a meta‐analysis previously conducted (Kambeitz et al., 2012). In the meta‐analysis, results suggested that BDNF Val66Met polymorphism accounts for a significant proportion of the interindividual variation in memory performance, including on both the structure and physiology of hippocampus (Kambeitz et al., 2012). However, it might be challenging to have a generalization regarding the influence of Val66Met polymorphism as there were different forms of memory that were reviewed. The hippocampus region where BDNF is highly expressed at is mainly responsible for consolidating short‐term memory into long‐term memory and the spatial memory (Bekinschtein et al., 2008). In fact, the distinct phenotypes of memory that were reviewed include but are not limited to episodic memory, associative memory, and working memory.

**Table 2:** List of reviewed studies showing the direction of association between Val66Met polymorphism and the respective neurocognitive domains for healthy individuals (*N* = 42)

BDNF genotype on cognitive domains (nondiseased states)						
References	Population	Composition of cases		Domain	Association	Direction of effect
		Val/Val (%)	Met carriers (%)			
Thow et al. (2017)	Healthy adults	322 (72.6)	121 (27.4)	M, EF	−	NA
Bombardier et al. (2016)	Healthy adults	49 (65.3)	26 (34.7)	M	−	NA
Wegman et al. (2016)	Healthy adults	18 (48.6)	19 (51.4)	M	−	NA
Wilkosc et al. (2016)	Healthy adults	*N* = 460		M, EF	−	NA
Thibeau et al. (2016)	Healthy adults	380 (65.9)	197 (34.1)	EF	−	NA
Chen et al. (2016)	Healthy adults	113 (27.0)	304 (73.0)	M	−	NA
Chen et al. (2015)	Healthy adults	25 (22.8)	85 (77.2)	M	−	NA
Ward et al. (2015)	Healthy adults	286 (66.1)	147 (33.9)	A, EF, VF, M	−	NA
Cherubin et al. (2014)	Healthy adults	261 (65.3)	139 (34.7)	A, EF	−	NA
Ward et al. (2014)	Healthy adults	282 (66.8)	140 (33.2)	M, EF	−	NA
Ghisletta et al. (2014)	Healthy adults	239 (66.0)	123 (34.0)	EF	−	NA
Montag et al. (2014)	Healthy adults	93 (67.0)	45 (33.0)	M	−	NA
Stuart et al. (2014)	Healthy adults	236 (65.6)	124 (34.4)	M	−	NA
Kennedy et al. (2015)	Healthy adults	79 (68.1)	37 (31.9)	M	−	NA
Liu et al. (2014)	Healthy adults	86 (26.1)	244 (73.9)	M	−	NA
Erickson et al. (2013)	Healthy adults	671 (65.0)	361 (35.0)	M, EF	−	NA
Freundlieb et al. (2012)	Healthy adults	22 (57.9)	16 (42.1)	A, EF, VF	−	NA
Dennis et al. (2011)	Healthy adults	11 (50.0)	11 (50.0)	A, EF, M	−	NA
Voineskos et al. (2011)	Healthy adults	41 (59.5)	28 (40.5)	M	−	NA
Beste et al. (2011)	Healthy adults	119 (56.4)	92 (43.6)	M	−	NA
Karnik et al. (2010)	Healthy adults	87 (67.4)	42 (32.6)	M, VF	−	NA
Cathomas et al. (2010)	Healthy adults	203 (61.0)	130 (39.0)	M	−	NA
Gatt et al. (2007)	Healthy adults	242 (64.7)	132 (35.3)	A	−	NA
Harris et al. (2006)	Healthy adults	589 (65.2)	315 (34.8)	EF, VF	−	NA
Huang et al. (2014)	Healthy adults	20 (22.2)	70 (77.8)	A, EF, VF, M	**+**	Met carriers (M)
Avgan et al. (2017)	Healthy adults	*N* = 181		M	**+**	Met carriers (M)
Jasinska et al. (2016)	Children	55 (67.9)	26 (32.1)	M	**+**	Val/Val (M)
Gosselin et al. (2016)	Healthy adults	79 (73.8)	28 (26.2)	M	**+**	Val/Val (M)
Canivet et al. (2015)	Elderly	118 (57.5)	87 (42.5)	M	**+**	Val/Val (M)
Lamb et al. (2015)	Healthy adults	53 (53.0)	47 (47.0)	M	**+**	Val/Val (M)
Gonzalez et al. (2016)	Healthy adults	*N* = 167		EF	**+**	Met carriers (EF)
Freundlieb et al. (2015)	Healthy adults	23 (60.5)	15 (39.5)	A, EF, VF, M	**+**	Met carriers (EF)
Gonzalez‐Giraldo et al. (2014)	Healthy adults	129 (77.0)	39 (23.0)	M	**+**	Met carriers (M)
Lim et al. (2013)	Healthy adults	107 (64.8)	58 (35.2)	EF, M, A	**+**	Val/Val (EF)
Yogeetha et al. (2013)	Healthy adults	113 (62.4)	68 (37.6)	M	**+**	Val/Val (M)
De Beaumont et al. (2013)	Healthy adults	80 (60.6)	52 (39.4)	M, EF, VF	**+**	Val/Val (M)
Alfimova et al. (2012)	Healthy adults	257 (64.0)	144 (36.0)	A, EF, VF, M	**+**	Met carriers (EF) (M)
Gajewski et al. (2011)	Healthy adults	79 (60.3)	52 (39.7)	M, EF, VF	**+**	Met carriers (M)
Richter‐Schmidinger et al. (2011)	Healthy adults	51 (37.8)	84 (62.2)	M	**+**	Val/Val (M)
Gong et al. (2009)	Healthy adults	219 (30.8)	582 (69.2)	M, EF, VF	**+**	Val/Val (M)
Schofield et al. (2009)	Healthy adults	282 (59.4)	193 (40.6)	M, A, EF, VF	**+**	Val/Val (M)
Raz et al. (2009)	Healthy adults	*N* = 189		M	**+**	Val/Val (M)
Miyajima et al. (2008)	Healthy adults	471 (55.3)	380 (44.7)	M	**+**	Val/Val (M)

*Notes*

A, attention and concentration; BDNF, brain‐derived neurotrophic factor; EF, executive function; M, memory; VF, verbal fluency. () denotes percentage of subjects showing the specified genotype. (−) shows the lack of an association between BDNF genotype with the cognitive domain, while (+) shows the presence of an association between BDNF genotype with the cognitive domain.

**Table 3:** List of reviewed studies showing the direction of the association between Val66Met polymorphism and the respective neurocognitive domains for clinical populations (*N* = 40)

BDNF genotype on cognitive domains (clinical population)						
References	Population	Composition of cases		Domain	Association	Direction of effect
		Val/Val (%)	Met carriers (%)			
Wei et al. (2017)	On GnRH agonist	29 (74.3)	10 (25.7)	M	−	NA
Lee et al. (2016)	Bipolar disorder	80 (22.5)	275 (77.5)	EF	−	NA
Lin et al. (2016)	Alzheimer	55 (29.5)	131 (70.5)	M, EF, A	−	NA
Gomar et al. (2016)	Alzheimer	153 (69.0)	69 (31.0)	M	−	NA
Mezquida et al. (2016)	Schizophrenia	124 (62.6)	74 (37.4)	M, EF, A	−	NA
Kim et al. (2015)	Dementia	300 (65.0)	160 (35.0)	M	−	NA
Bialecka et al. (2014)	Parkinson	176 (72.1)	68 (27.9)	M, EF	−	NA
Fera et al. (2013)	Sclerosis	12 (46.2)	14 (53.8)	M, EF	−	NA
Martinho et al. (2012)	Psychosis	88 (67.7)	42 (32.3)	M, VF	−	NA
Molendijk et al. (2012)	Depression	82 (65.1)	44 (34.9)	M	−	NA
da Rocha et al. (2011)	OCD	82 (67.2)	40 (32.8)	A, EF	−	NA
Swardfager et al. (2011)	Cardiovascular	55 (65.5)	29 (34.5)	A, EF, M	−	NA
Nagata et al. (2011)	Alzheimer	45 (26.6)	124 (73.4)	EF	−	NA
Yu et al. (2008)	Brain Injury	31 (31.3)	68 (68.7)	M, EF, VF, A	−	NA
Oroszi et al. (2006)	SLE	46 (78.0)	13 (22.0)	M, A, EF	−	NA
Strauss et al. (2004)	Depression	43 (69.3)	19 (30.7)	M	−	NA
Zhang et al. (2016)	Schizophrenia	*N* = 1,887		A, M	−	N/A
Ho et al. (2007)	Schizophrenia	74 (62.2)	45 (37.8)	M	−	N/A
Nagata et al. (2012)	Alzheimer	41 (28.1)	105 (71.9)	EF	**+**	Met/Met (EF)
Zhang et al. (2012)	Schizophrenia	175 (27.0)	474 (73.0)	A	**+**	Val/Val (A)
Ho et al. (2006)	Schizophrenia	182 (62.1)	111 (37.9)	A, EF, M	**+**	Val/Val (M)
Lim et al. (2016)	Alzheimer	95 (34.7)	179 (65.3)	M	**+**	Val/Val (M)
Kim et al. (2016)	Schizophrenia	102 (76.7)	31 (23.3)	M, EF, A	**+**	Met carriers (M, EF, A)
Narayanan et al. (2016)	pTBI	16 (33.3)	32 (66.7)	M, EF, A	**+**	Val/Val (M, EF, A)
Cao et al. (2016)	Depression (Bipolar)	60 (74.1)	21 (25.9)	M	**+**	Val/Val (M)
Altmann et al. (2016)	Parkinson	117 (66.8)	58 (33.2)	EF	**+**	Val/Val (EF)
Ng et al. (2016)	Postchemotherapy	38 (26.2)	107 (73.8)	EF, VF	**+**	Met carriers (EF, VF)
Lee et al. (2015)	Alzheimer	16 (15.1)	90 (84.9)	EF	**+**	Val/Val (EF)
van der Kolk et al. (2015)	Parkinson	230 (60.0)	154 (40.0)	EF, VF	**+**	Met carriers (EF)
Yin et al. (2015)	Depression	8 (30.8)	18 (69.2)	EF	**+**	Met carriers (EF)
Szabo et al. (2013)	Cardiovascular	77 (70.0)	33 (30.0)	M, A, EF, VF	**+**	Met carriers (M, A, EF)
Barbey et al. (2014)	Brain injury	97 (62.2)	59 (37.8)	EF	**+**	Met carriers (EF)
Aas et al. (2013)	Psychosis	170 (68.3)	79 (31.7)	M, EF, VF	**+**	Val/Val (M)
Tukel et al. (2012)	OCD	23 (23.0)	77 (77.0)	M, A. EF, VF	**+**	Val/Val (M, EF)
McAllister et al. (2012)	Brain injury	*N* = 75		M, EF	**+**	Met carriers (EF)
Rybakowski et al. (2006)	Schizophrenia	84 (65.1)	45 (34.9)	EF	**+**	Val/Val (EF)
Tan et al. (2005)	Schizophrenia	28 (26.0)	80 (74.0)	M	**+**	Val/Val (M)
Egan et al. (2003)	Schizophrenia	138 (68.0)	65 (32.0)	M, EF	**+**	Val/Val (M)
Rybakowski et al. (2003)	Bipolar	44 (81.5)	10 (18.5)	EF, M	**+**	Val/Val (EF)

*Notes*

A, attention and concentration, BDNF, brain‐derived neurotrophic factor; EF, executive function; M, memory; VF, verbal fluency. () denotes percentage of subjects showing the specified genotype. (−) shows the lack of an association between BDNF genotype with the cognitive domain, while (+) shows the presence of an association between BDNF genotype with the cognitive domain.

The various types of memory would require an independent measure of their own and are likely to be influenced by BDNF polymorphism to varying extents. In order to make reliable and reasonable comparisons across studies, the measures for memory tests could have been standardized or be matched to a defined memory. To illustrate our point, while one study reported findings that Val/Val homozygotes were found to perform better in spatial memory compared to the other genotypes, they scored worse on measure of visual memory (Yogeetha et al., 2013). In another study looking at schizophrenia population, genotypes of Val/Val homozygotes were found to have better verbal memory and logical episodic memory (Zhang et al., 2012). While it appears that Val66Met polymorphism confers better verbal memory, it is noteworthy that our reviewed studies did not show an association with verbal fluency (Table 1). This indicates that there may be some specificity of association of Val66Met polymorphisms with the respective cognitive domains.

Another objective measure worth exploring as a basis of comparison across studies is to conduct neuroimaging analysis such as fMRI to assess actual neural activation in brain activity. A meta‐analysis conducted found no significant association between BDNF Val66Met polymorphism and hippocampal volumes in healthy humans, with hippocampal volumes being of interest because it is a well‐established brain region that has an important role in learning and memory. However, the findings observed that Met carriers had slightly smaller hippocampal volumes than Val/Val homozygotes (Harrisberger et al., 2014). Given the current advancement in neuroimaging techniques, it might be worth looking at the exact brain regions that are activated when correlating with cognitive function. This would enhance our mechanistic understanding behind the biological mechanism.

With regards to the attention/concentration domain, it was interesting to note that positive associations were identified only in cohorts with specific disease states. The findings from one study favors Met carriers, suggesting that Met allele favorably interacts with cognitive changes associated with the cardiovascular disease or that cardiovascular diseases may have affected circulating levels of BDNF, given that BDNF is permeable to the blood–brain barrier (Szabo et al., 2013). Another study finds that Met carriers, regardless of whether they were healthy controls or schizophrenic, performed worse than genotypes of Val/Val homozygotes in the attention/concentration domains (Zhang et al., 2012). It is postulated that hippocampal neurons that contain Met allele showed less depolarization‐induced BDNF secretion, resulting in reduced activity‐dependent BDNF release and poorer hippocampal‐mediated function (Zhang et al., 2012).

In reviewing the studies on the domain of executive function domain, 11 studies favored Met carriers compared to studies that favor Val/Val homozygotes. It was stated that the accumulating evidence in human lesion patients on Val66Met polymorphism has shown that Met allele exerts a protective effect for executive function in a study on patients who had traumatic brain injury (Barbey et al., 2014). Similarly, another study showed that Met carriers showed a reduced relationship between cognitive reserve and executive function although it is rationalized that the lowered functional capacity can be attributed to the negative intrinsic effect of BDNF Met polymorphism. Consequentially, cognitive reserve was reported to account for up to 8.5% of the variance in executive function in Val/Val homozygotes (Ward et al., 2015).

There are suggestions that certain disease states could be preferentially influenced by the impact of the Met allele on neurocognition. The presence as well as the types of neurological conditions such as Alzheimer’s disease, Parkinson’s disease, traumatic brain injury, and depression in the studied population may influence whether Met allele exerts a neuroprotective effect. For the population that has schizophrenia, most studies were inclined toward favoring Val/Val homozygotes (Egan et al., 2003; Rybakowski et al., 2003, 2006; Tan et al., 2005; Zhang et al., 2012).

In some instances, poorer neurocognition could be attributed to neurodegenerative diseases itself rather than BDNF polymorphism, confounding the actual extent of effect from the BDNF genotype. Disease states which affect cognitive function may mask or worsen the effect of genetic polymorphism on cognition. While BDNF polymorphism was not significantly associated with serum BDNF, this polymorphism is believed to influence intracellular sorting of BDNF, resulting in decreased synaptic plasticity (Bath & Lee, 2006; Binder & Scharfman, 2004). The biological function of pro‐BDNF is also not limited in being an inactive precursor with some suggesting that it may have an antagonistic effect (Bath & Lee, 2006; Binder & Scharfman, 2004). The overall physiological functions may be better correlated by a balance of both pro and mature BDNF, so measuring BDNF as a ratio of pro: mature forms might be a more accurate reflection of BDNF effect in relation to brain disorders. There are also variations attributed to the source of BDNF as levels differ from plasma to serum samples. Our study posits that, without the direct quantification of circulating BDNF, it might be difficult to evaluate the association between BDNF genotype and cognitive function.

In a study involving patients with Alzheimer’s disease, Met carriers were reported to have better executive function. It was suggested that Met carriers are protected from the hippocampus cortical atrophy or subcortical tract changes, leading to them having a decreased risk of Alzheimer’s and improved performance in domain related to executive function (Nagata et al., 2012). However, it remains unclear if the hippocampal atrophy is attributed to neuropsychiatric disorders or the extent of contribution genetic polymorphism has on its effect.

Conversely, in another study targeting at patients with high level of beta‐amyloid (Aβ), it was reported that there is greater hippocampal atrophy found in Met carriers. Met carriers were found to show a significant decline in executive functions compared to genotypes of Val/Val homozygotes over a 36‐month period instead (Lim et al., 2013). Met carriers were also found with worse memory performance in an Alzheimer’s population, with deleterious effects of Aβ on memory found to be greater in Met carriers (Lim et al., 2016). This suggests that high Aβ level might have been a necessary condition for cognitive decline in this context and that BDNF Val66Met polymorphism has a downstream moderation of the effect of Aβ in executive function. The implication of these findings is that cognitive influence of BDNF polymorphism might not necessarily be related to its effect on brain region.

Old age could also be another factor that determines the direction of effect BDNF Val66Met polymorphism has on neurocognition as aging is often accompanied by decrease in hippocampus volume which is associated with decline in episodic memory (Harrisberger et al., 2014). Some studies have demonstrated that Met carriers are more protected during aging than Val/Val homozygotes when it comes to cognitive function such as executive functions that involve the prefrontal cortex. For some aspects of memory, Met carriers were found to be performed poorly in associative memory, regardless of age. BDNF Val66Met polymorphism exerts an influence regardless of age on subjective and associative memory while may exacerbate age‐related differences in prospective memory (Kennedy et al., 2015).

Children aged between 6 and 10 who are Val/Val homozygotes were found to outperform Met carriers on tasks that have a strong memory component and exert lesser effort in neural activation compared to Met carriers on fMRI analysis (Jasinska et al., 2016). Yet in another study examining the influence of BDNF Val66Met polymorphism on behavioral outcome of healthy young adults between 20 and 30 years old, Met carriers tend toward better performance. It is implied that the polymorphism can be an advantage or a disadvantage depending on the tasks examined as each genotype use slightly different brain activation pattern as some performance‐related tasks may be more dependent on BDNF (Dennis et al., 2011). It is also plausible that BDNF Val66Met differences on neurocognition may be dependent on different developmental stages between neonates, children, adolescents, and the elderly. To address this, there could be longitudinal studies that assess for the domains of interest over time, for comparison in the future.

Given that the included studies vary greatly in terms of study designs, sample sizes, and neuropsychological batteries that were used to assess the subjects of interest, there are several limitations in this systematic review. The lack of consistent standards throughout could explain why results were not reproducible across studies. In addition, the small sample size of some studies, especially those with stricter definitions may also lack statistical power in their analysis. Moreover, our study categorized the genotypes collectively as Met carriers to compare against Val/Val homozygotes, while some studies may further differentiate the Met carriers into individual Met/Val and Met/Met genotypes for comparison. The association reported might also be influenced by ethnicity‐ a variable that has not been adjusted for. It has been observed that different ethnic groups showed differences in genotypic frequency of the Val66Met polymorphism, with Caucasians appearing more susceptible to effects of having Met alleles. The derivative Met allele ranges from 0% in Africa to up to 60% in Asia, and a 17% frequency in Caucasians (Mandelman & Grigorenko, 2012), entailing that patient groups may need to be matched to make a more representative comparison.

Moving forward, it might be interesting to note how BDNF polymorphism might have a role in cognitive function especially in terms of memory. If there is indeed a significant association, genetic testing could be conducted early on patient groups to segregate them to according to genotypes of the Val/Val homozygotes or Met carriers for active interventions. If a correlation can be found with disease states that are neurological in nature and which collaborate with neuroimaging findings, it may lend strength to the argument for preemptive scanning to be carried out at the affected brain regions. Our reviewed studies also suggest that the influence of genetics on cognition could become more apparent under certain disease states or aging. While Met allele is hypothesized to negatively affect intracellular trafficking and activity‐dependent secretion of BDNF and is expected to cause poorer neurocognition performance, there is also an interplay of factors between gene, environment, and disease state in determining the direction of effect. For instance, there has been a cross‐sectional study which found that the association between physical activity and episodic memory was mediated by BDNF polymorphism, as Val/Val homozygotes were found to perform significantly better than Met carriers if they were active in physical activity, showing how that environmental factor can also be a consideration (Canivet et al., 2015).

Overall, consolidated results from our reviewed studies showed that BDNF polymorphisms’ relationship with cognition might be more complex than previously thought. More future studies could delve into the correlation between the gene loci and cognitive domains.

## 5 CONCLUSION

Across the four domains that were studied, approximately half of the identified studies reported an association between Val66Met polymorphism with neurocognitive domains. In particular, Val/Val homozygotes performed better in tasks related to the memory domain. On the other hand, Met carriers appear to give rise to improved neurocognitive performances in terms of executive function. The results from the studies focusing on the domain of verbal fluency and attention/concentration were less conclusive. While more studies might be needed in the future with a more clearly defined cohort and comorbidities being adjusted for, our findings suggest that Val66Met polymorphism may be associated with the cognitive domains of executive function and memory domains although the effect of Met allele remains to be clearly established. More consistent replicated findings of the Val66Met polymorphism with neurocognition are required in the future to extend its clinical relevance.

## CONFLICT OF INTEREST

None declared.

## Footnotes

1 Counted as proportion of studies out of total unique studies favoring

## References

[brb31009-bib-0001] Aas, M. , Haukvik, U. K. , Djurovic, S. , Bergmann, Ø. , Athanasiu, L. , Tesli, M. S. , … Melle, I. (2013). BDNF val66met modulates the association between childhood trauma, cognitive and brain abnormalities in psychoses. Progress in Neuro‐Psychopharmacology and Biological Psychiatry, 46, 181–188. 10.1016/j.pnpbp.2013.07.008 23876786

[brb31009-bib-0002] Acheson, A. , Conover, J. , Fandl, J. , DeChiara, T. M. , Russell, M. , Thadani, A. , … Lindsya, R. M. (1995). A BDNF autocrine loop in adult sensory neurons prevents cell death. Nature, 375, 450 10.1038/374450a0 7700353

[brb31009-bib-0003] Adachi, N. , Numakawa, T. , Richards, M. , Nakajima, S. , & Kunugi, H. (2014). New insight in expression, transport, and secretion of brain‐derived neurotrophic factor: Implications in brain‐related diseases. World Journal of Biological Chemistry, 5(4), 409–428. 10.4331/wjbc.v5.i4.409 25426265 PMC4243146

[brb31009-bib-0004] Alfimova, M. V. , Korovaitseva, G. I. , Lezheiko, T. V. , & Golimbet, V. E. (2012). Effect of BDNF Val66Met polymorphism on normal variability of executive function. Bulletin of Experimental Biology and Medicine, 152(5), 606–609. 10.1007/s10517-012-1587-x 22803145

[brb31009-bib-0005] Altmann, V. , Schumacher‐Schuh, A. F. , Rieck, M. , Callegari‐Jacques, S. M. , Rieder, C. R. , & Hutz, M. H. (2016). Val66Met BDNF polymorphism is associated with Parkinson’s disease cognitive impairment. Neuroscience Letters, 615, 88–91. 10.1016/j.neulet.2016.01.030 26806863

[brb31009-bib-0006] Avgan, N. , Sutherland, H. G. , Spriggens, L. K. , Yu, C. , Ibrahim, O. , Bellis, C. , … Griffiths, L. R. (2017). DNF variants may modulate long‐term visual memory performance in a healthy cohort. International Journal of Molecular Sciences, 18(3), pii: E655 10.3390/ijms18030655 PMC5372667 28304362

[brb31009-bib-0007] Barbey, A. K. , Colom, R. , Paul, E. , Forbes, C. , Krueger, F. , Goldman, D. , & Grafman, J. (2014). Preservation of general intelligence following traumatic brain injury: Contributions of the Met66 brain‐derived neurotrophic factor. PLoS ONE, 9(2), e88733 10.1371/journal.pone.0088733 24586380 PMC3935849

[brb31009-bib-0008] Bath, K. , & Lee, F. (2006). Variant BDNF (Val66Met) impact on brain structure and function. Cognitive, Affective & Behavioral Neuroscience, 6(1), 79–85. 10.3758/CABN.6.1.79 16869232

[brb31009-bib-0009] Bekinschtein, P. , Cammarota, M. , & Katche, C. (2008). BDNF is essential to promote persistence of long‐term memory storage. Proceedings of the National Academy of Sciences of the United States of America, 105, 2711–2716. 10.1073/pnas.0711863105 18263738 PMC2268201

[brb31009-bib-0010] Beste, C. , Schneider, D. , Epplen, J. T. , & Arning, L. (2011). The functional BDNF Val66Met polymorphism affects functions of pre‐attentive visual sensory memory processes. Neuropharmacology, 60(2–3), 467–471. 10.1016/j.neuropharm.2010.10.028 21056046

[brb31009-bib-0011] Bialecka, M. , Kurzawski, M. , Roszmann, A. , Robowski, P. , Sitek, E. J. , Honczarenko, K. , … Sławek, J. (2014). BDNF G196A (Val66Met) polymorphism associated with cognitive impairment in Parkinson’s disease. Neuroscience Letters, 561, 86–90. 10.1016/j.neulet.2013.12.051 24394906

[brb31009-bib-0012] Binder, D. , & Scharfman, H. (2004). Brain‐derived neurotrophic factor. Growth Factors, 22(3), 123–131. 10.1080/08977190410001723308 15518235 PMC2504526

[brb31009-bib-0013] Bombardier, A. , Beauchemin, M. , Gosselin, N. , Poirier, J. , & De Beaumont, L. (2016). Altered episodic memory in introverted young adults carrying the BDNFMet allele. International Journal of Molecular Sciences, 17(11), pii: E1886 10.3390/ijms17111886 PMC5133885 27845759

[brb31009-bib-0014] Canivet, A. , Albinet, C. T. , Andre, N. , Pylouster, J. , Rodríguez‐Ballesteros, M. , Kitzis, A. , & Audiffren, M. (2015). Effects of BDNF polymorphism and physical activity on episodic memory in the elderly: A cross sectional study. European Review of Aging and Physical Activity, 12, 15 10.1186/s11556-015-0159-2 26865879 PMC4748321

[brb31009-bib-0015] Cao, B. , Bauer, I. E. , Sharma, A. N. , Mwangi, B. , Frazier, T. , Lavagnino, L. , … Soares, J. C. (2016). Reduced hippocampus volume and memory performance in bipolar disorder patients carrying the BDNF val66met met allele. Journal of Affective Disorders, 198, 198–205. 10.1016/j.jad.2016.03.044 27018938 PMC5214589

[brb31009-bib-0016] Cathomas, F. , Vogler, C. , Euler‐Sigmund, J. C. , de Quervain, D. J. , & Papassotiropoulos, A. (2010). Fine‐mapping of the brain‐derived neurotrophic factor (BDNF) gene supports an association of the Val66Met polymorphism with episodic memory. International Journal of Neuropsychopharmacology, 13(8), 975–980. 10.1017/S1461145710000519 20482942

[brb31009-bib-0017] Chen, C. C. , Chen, C. J. , Wu, D. , Chi, N. F. , Chen, P. C. , Liao, Y. P. , … Hu, C. J. (2015). BDNF Val66Met polymorphism on functional MRI during n‐back working memory tasks. Medicine (Baltimore), 94(42), e1586 10.1097/MD.0000000000001586 26496261 PMC4620795

[brb31009-bib-0018] Chen, W. , Chen, C. , Xia, M. , Wu, K. , Chen, C. , He, Q. , … Dong, Q. (2016). Interaction effects of BDNF and COMT genes on resting‐state brain activity and working memory. Frontiers in Human Neuroscience, 10, 540. 27853425 10.3389/fnhum.2016.00540 PMC5091010

[brb31009-bib-0019] Cherubin, N. , Das, D. , Tan, X. , Bielak, A. , Eastel, S. , & Anstey, K. J. (2014). Cognitive ability, intraindividual variability and common genetic variants of catechol‐*O*‐methyltransferase and brain‐derived neurotrophic factor: A longitudinal study in a population‐based sample of older adults. Psychology and Aging, 29(2), 393–403. 24956006 10.1037/a0035702

[brb31009-bib-0020] De Beaumont, L. , Fiocco, A. J. , Quesnel, G. , Lupien, S. , & Poirier, J. (2013). Altered declarative memory in introverted middle‐aged adults carrying the BDNF val66met allele. Behavioral Brain Research, 253, 152–156. 10.1016/j.bbr.2013.07.002 23835043

[brb31009-bib-0021] Dennis, N. A. , Cabeza, R. , Need, A. C. , Waters‐Metenier, S. , Goldstein, D. B. , & LaBar, K. S. (2011). Brain‐derived neurotrophic factor val66met polymorphism and hippocampal activation during episodic encoding and retrieval tasks. Hippocampus, 21(9), 980–989. 20865733 10.1002/hipo.20809 PMC3010486

[brb31009-bib-0022] Egan, M. , Kojima, M. , & Callicott, J. (2003). The BDNF Val66met polymorphism affects activity‐dependent secretion of BDNF and human memory and hippocampal function. Cell, 112(2), 257–269. 10.1016/S0092-8674(03)00035-7 12553913

[brb31009-bib-0023] Erickson, K. I. , Banducci, S. E. , Weinstein, A. M. , Macdonald, A. W. 3rd , Ferrell, R. E. , Halder, I. , … Manuck, S. B. (2013). The brain‐derived neurotrophic factor Val66Met polymorphism moderates an effect of physical activity on working memory performance. Psychological Science, 24(9), 1770–1779. 10.1177/0956797613480367 23907543 PMC3947596

[brb31009-bib-0024] Fera, F. , Passamonti, L. , Cerasa, A. , Gioia, M. C. , Liguori, M. , Manna, I. , … Quattrone, A. (2013). The BDNF Val66Met polymorphism has opposite effects on memory circuits of multiple sclerosis patients and controls. PLoS ONE, 8(4), e61063 10.1371/journal.pone.0061063 23593393 PMC3623818

[brb31009-bib-0025] Freundlieb, N. , Backhaus, W. , Bruggemann, N. , Gerloff, C. , Klein, C. , Pinnschmidt, H. O. , & Hummel, F. C. (2015). Differential effects of BDNF val(66)met in repetitive associative learning paradigms. Neurobiology of Learning and Memory, 123, 11–17. 10.1016/j.nlm.2015.04.010 25933507

[brb31009-bib-0026] Freundlieb, N. , Philipp, S. , Schneider, S. A. , Brüggemann, N. , Klein, C. , Gerloff, C. , & Hummel, F. C. (2012). No association of the BDNF val66met polymorphism with implicit associative vocabulary and motor learning. PLoS ONE, 7(11), e48327 10.1371/journal.pone.0048327 23152767 PMC3496723

[brb31009-bib-0027] Gajewski, P. D. , Hengstler, J. G. , Golka, K. , Falkenstein, M. , & Beste, C. (2011). The Met‐allele of the BDNF Val66Met polymorphism enhances task switching in elderly. Neurobiology of Aging, 32(12), 2327.e7–e19. 10.1016/j.neurobiolaging.2011.06.010 21803453

[brb31009-bib-0028] Gatt, J. M. , Clark, C. R. , Kemp, A. H. , Liddell, B. J. , Dobson‐Stone, C. , Kuan, S. A. , … Williams, L. M. (2007). A genotype–endophenotype–phenotype path model of depressed mood: Integrating cognitive and emotional markers. Journal of Integrative Neuroscience, 06(01), 75–104. 10.1142/S0219635207001398 17472225

[brb31009-bib-0029] Ghisletta, P. , Backman, L. , Bertram, L. , Brandmaier, A. M. , Gerstorf, D. , Liu, T. , & Lindenberger, U. (2014). The Val/Met polymorphism of the brain‐derived neurotrophic factor (BDNF) gene predicts decline in perceptual speed in older adults. Psychology and Aging, 29(2), 384–392. 10.1037/a0035201 24660789

[brb31009-bib-0030] Gomar, J. J. , Conejero‐Goldberg, C. , Huey, E. D. , Davies, P. , & Goldberg, T. E. (2016). Alzheimer’s disease neuroimaging I. Lack of neural compensatory mechanisms of BDNF val66met met carriers and APOE E4 carriers in healthy aging, mild cognitive impairment, and Alzheimer’s disease. Neurobiology of Aging, 39, 165–173. 10.1016/j.neurobiolaging.2015.12.004 26923413 PMC9969539

[brb31009-bib-0031] Gong, P. , Zheng, A. , Chen, D. , Ge, W. , Lv, C. , Zhang, K. , … Zhang, F. (2009). Effect of BDNF Val66Met polymorphism on digital working memory and spatial localization in a healthy Chinese Han population. Journal of Molecular Neuroscience, 38(3), 250–256. 10.1007/s12031-009-9205-8 19424874

[brb31009-bib-0032] Gonzalez, G. , Mueller, S. , Adan, A. , Rojas, J. , Piper, B. , & Forero, D. (2016). BDNF Val66Met is associated with performance in a computerized visual‐motor tracking test in healthy adults. Motor Control, 20, 122–134. 10.1123/mc.2014-0075 25823467

[brb31009-bib-0033] Gonzalez‐Giraldo, Y. , Rojas, J. , Novoa, P. , Mueller, S. T. , Piper, B. J. , Adan, A. , & Forero, D. A. (2014). Functional polymorphisms in BDNF and COMT genes are associated with objective differences in arithmetical functioning in a sample of young adults. Neuropsychobiology, 70(3), 152–157. 10.1159/000366483 25358337

[brb31009-bib-0034] Gosselin, N. , De Beaumont, L. , Gagnon, K. , Baril, A. A. , Mongrain, V. , Blais, H. , … Carrier, J. (2016). BDNF Val66Met polymorphism interacts with sleep consolidation to predict ability to create new declarative memories. Journal of Neuroscience, 36(32), 8390–8398. 10.1523/JNEUROSCI.4432-15.2016 27511011 PMC5811258

[brb31009-bib-0035] Harris, S. E. , Fox, H. , Wright, A. F. , Hayward, C. , Starr, J. M. , Whalley, L. J. , & Deary, I. J. (2006). The brain‐derived neurotrophic factor Val66Met polymorphism is associated with age‐related change in reasoning skills. Molecular Psychiatry, 11(5), 505–513. 10.1038/sj.mp.4001799 16446742

[brb31009-bib-0036] Harrisberger, F. , Spalek, K. , Smieskova, R. , Schmidt, A. , Coynel, D. , Milnik, A. , … Borgwardt, S. (2014). The association of the BDNF Val66Met polymorphism and the hippocampal volumes in healthy humans: A joint meta‐analysis of the published and new data. Neuroscience and Biobehavioral Reviews, 42, 267–278. 10.1016/j.neubiorev.2014.03.011 24674929

[brb31009-bib-0037] Ho, B. , Andreasen, N. , Dawson, J. , & Wassink, T. (2007). Association between brain‐derived neurotrophic factor Val66met gene polymorphism and progressive brain volume changes in schizophrenia. American Journal of Psychiatry, 164(12), 1890–1899. 10.1176/appi.ajp.2007.05111903 18056245 PMC3062255

[brb31009-bib-0038] Ho, B. , Mileve, P. , O’Leary, D. , Librant, A. , Andreasen, N. , & Wassink, T. (2006). Cognitive and magnetic resonance imaging brain morphometric correlates of brain derived neurotrophic factor Val66Met polymorphism in patients with schizophrenia and healthy volunteers. Archives of General Psychiatry, 63, 731–740. 10.1001/archpsyc.63.7.731 16818862 PMC3065118

[brb31009-bib-0039] Huang, C. C. , Liu, M. E. , Chou, K. H. , Yang, A. C. , Hung, C. C. , Hong, C. J. , … Lin, C. P. (2014). Effect of BDNF Val66Met polymorphism on regional white matter hyperintensities and cognitive function in elderly males without dementia. Psychoneuroendocrinology, 39, 94–103. 10.1016/j.psyneuen.2013.09.027 24275008

[brb31009-bib-0040] Huang, E. , & Reichardt, L. (2001). Neurotrophins: Roles in neuronal development and function. Annual Review of Neuroscience, 24, 677–736. 10.1146/annurev.neuro.24.1.677 PMC2758233 11520916

[brb31009-bib-0041] Jasinska, K. K. , Molfese, P. J. , Kornilov, S. A. , Mencl, W. E. , Frost, S. J. , Lee, M. , … Landi, N. (2016). The BDNF Val66Met polymorphism influences reading ability and patterns of neural activation in children. PLoS ONE, 11(8), e0157449 10.1371/journal.pone.0157449 27551971 PMC4995017

[brb31009-bib-0042] Kambeitz, J. P. , Bhattacharyya, S. , Kambeitz‐Ilankovic, L. M. , Valli, I. , Collier, D. A. , & McGuire, P. (2012). Effect of BDNF val(66)met polymorphism on declarative memory and its neural substrate: A meta‐analysis. Neuroscience and Biobehavioral Reviews, 36(9), 2165–2177. 10.1016/j.neubiorev.2012.07.002 22813992

[brb31009-bib-0043] Karnik, M. S. , Wang, L. , Barch, D. M. , Morris, J. C. , & Csernansky, J. G. (2010). BDNF polymorphism rs6265 and hippocampal structure and memory performance in healthy control subjects. Psychiatry Research, 178(2), 425–429. 10.1016/j.psychres.2009.09.008 20493546 PMC2950008

[brb31009-bib-0044] Kennedy, K. M. , Reese, E. D. , Horn, M. M. , Sizemore, A. N. , Unni, A. K. , Meerbrey, M. E. , … Rodrigue, K. M. (2015). BDNF val66met polymorphism affects aging of multiple types of memory. Brain Research, 1612, 104–117. 10.1016/j.brainres.2014.09.044 25264352 PMC4377126

[brb31009-bib-0045] Kim, A. , Fagan, A. , Goate, A. , Benzinger, T. , Morris, J. , & Head, D. (2015). Lack of an association of BDNF Val66Met polymorphism and plasma BDNF with hippocampal volume and memory. Cognitive, Affective & Behavioral Neuroscience, 15(3), 625–643. 10.3758/s13415-015-0343-x PMC4529376 25784293

[brb31009-bib-0046] Kim, S. W. , Lee, J. Y. , Kang, H. J. , Kim, S. Y. , Bae, K. Y. , Kim, J. M. , … Yoon, J. S. (2016). Gender‐specific associations of the brain‐derived neurotrophic factor Val66Met polymorphism with neurocognitive and clinical features in schizophrenia. Clinical Psychopharmacology and Neuroscience, 14(3), 270–278. 10.9758/cpn.2016.14.3.270 27489381 PMC4977808

[brb31009-bib-0047] van der Kolk, N. M. , Speelman, A. D. , van Nimwegen, M. , Kessels, R. P. , IntHout, J. , Hakobjan, M. , … van de Warrenburg, B. P. (2015). BDNF polymorphism associates with decline in set shifting in Parkinson’s disease. Neurobiology of Aging, 36(3), 1605.e1–e6 10.1016/j.neurobiolaging.2014.08.023 25444596

[brb31009-bib-0048] Lamb, Y. N. , Thompson, C. S. , McKay, N. S. , Waldie, K. E. , & Kirk, I. J. (2015). The brain‐derived neurotrophic factor (BDNF) val66met polymorphism differentially affects performance on subscales of the Wechsler Memory Scale – Third Edition (WMS‐III). Frontiers in Psychology, 6, 1212. 26347681 10.3389/fpsyg.2015.01212 PMC4538220

[brb31009-bib-0049] Lee, S. J. , Baek, J. H. , & Kim, Y. H. (2015). Brain‐derived neurotrophic factor is associated with cognitive impairment in elderly Korean individuals. Clinical Psychopharmacology and Neuroscience, 13(3), 283–287. 10.9758/cpn.2015.13.3.283 26598587 PMC4662173

[brb31009-bib-0050] Lee, S. Y. , Wang, T. Y. , Chen, S. L. , Chang, Y. H. , Chen, P. S. , Huang, S. Y. , … Chen, C. S. (2016). The correlation between plasma brain‐derived neurotrophic factor and cognitive function in bipolar disorder is modulated by the BDNF Val66Met polymorphism. Scientific Reports, 6, 37950 10.1038/srep37950 27905499 PMC5131343

[brb31009-bib-0051] Lim, Y. Y. , Hassenstab, J. , Cruchaga, C. , Goate, A. , Fagan, A. M. , Benzinger, T. L. , … Dominantly Inherited Alzheimer Network (2016). BDNF Val66Met moderates memory impairment, hippocampal function and tau in preclinical autosomal dominant Alzheimer’s disease. Brain, 139(Pt 10), 2766–2777. 10.1093/brain/aww200 27521573 PMC5815565

[brb31009-bib-0052] Lim, Y. Y. , Villemagne, V. , Laws, S. , Ames, D. , Pietrzak, R. , & Ellis, K. (2013). BDNF Val66Met, Aβ amyloid, and cognitive decline in preclinical Alzheimer’s disease. Neurobiology of Aging, 34(11), 2457–2464. 10.1016/j.neurobiolaging.2013.05.006 23769397

[brb31009-bib-0053] Lin, P. H. , Tsai, S. J. , Huang, C. W. , Mu‐En, L. , Hsu, S. W. , Lee, C. , … Chang, C. (2016). Dose‐dependent genotype effects of BDNF Val66Met polymorphism on default mode network in early stage Alzheimer’s disease. Oncotarget, 7(34), 54200–54214. 27494844 10.18632/oncotarget.11027 PMC5342335

[brb31009-bib-0054] Liu, M. E. , Huang, C. C. , Chen, M. H. , Yang, A. C. , Tu, P. C. , Yeh, H. L. , … Tsai, S. J. (2014). Effect of the BDNF Val66Met polymorphism on regional gray matter volumes and cognitive function in the Chinese population. Neuromolecular Medicine, 16(1), 127–136. 10.1007/s12017-013-8265-7 24366608

[brb31009-bib-0055] Mandelman, S. D. , & Grigorenko, E. L. (2012). BDNF Val66Met and cognition: All, none, or some? A meta‐analysis of the genetic association. Genes, Brain, and Behavior, 11(2), 127–136. 10.1111/j.1601-183X.2011.00738.x PMC3268899 21980924

[brb31009-bib-0056] Martinho, E. M. Jr , Michelon, L. , Ayres, A. M. , Scazufca, M. , Menezes, P. R. , … Schaufelberger, M. S. (2012). Filho G BDNF gene polymorphism, cognition and symptom severity in a brazilian population‐based sample of first‐episode psychosis subjects. Revista Brasileira de Psiquiatria, 34, 219–232. 10.1016/j.rbp.2012.06.004 23429848

[brb31009-bib-0057] Matsuo, K. , Walss‐Bass, C. , Nery, F. G. , Nicoletti, M. A. , Hatch, J. P. , Frey, B. N. , … Soares, J. C. (2009). Neuronal correlates of brain‐derived neurotrophic factor Val66Met polymorphism and morphometric abnormalities in bipolar disorder. Neuropsychopharmacology, 34(8), 1904–1913. 10.1038/npp.2009.23 19295510

[brb31009-bib-0058] McAllister, T. W. , Tyler, A. L. , Flashman, L. A. , Rhodes, C. H. , McDonald, B. C. , Saykin, A. J. , … Moore, J. H. (2012). Polymorphisms in the brain‐derived neurotrophic factor gene influence memory and processing speed one month after brain injury. Journal of Neurotrauma, 29(6), 1111–1118. 10.1089/neu.2011.1930 22188054 PMC3325555

[brb31009-bib-0059] Mezquida, G. , Penades, R. , Cabrera, B. , Savulich, G. , Lobo, A. , González‐Pinto, A. , … PEPs group . (2016). Association of the brain‐derived neurotrophic factor Val66Met polymorphism with negative symptoms severity, but not cognitive function, in first‐episode schizophrenia spectrum disorders. European Psychiatry, 38, 61–69. 10.1016/j.eurpsy.2016.04.011 27668551

[brb31009-bib-0060] Miyajima, F. , Ollier, W. , Mayes, A. , Jackson, A. , Thacker, N. , Rabbitt, P. , … Payton, A. (2008). Brain‐derived neurotrophic factor polymorphism Val66Met influences cognitive abilities in the elderly. Genes, Brain, and Behavior, 7(4), 411–417. 10.1111/j.1601-183X.2007.00363.x 17973920

[brb31009-bib-0061] Molendijk, M. L. , van Tol, M. J. , Penninx, B. W. , van der Wee, N. J. , Aleman, A. , Veltman, D. J. , … Elzinga, B. M. (2012). BDNF val66met affects hippocampal volume and emotion‐related hippocampal memory activity. Translational Psychiatry, 2, e74 10.1038/tp.2011.72 22832736 PMC3309548

[brb31009-bib-0062] Montag, C. , Felten, A. , Markett, S. , Fischer, L. , Winkel, K. , Cooper, A. , & Reuter, M. (2014). The role of the BDNF Val66Met polymorphism in individual differences in long‐term memory capacity. Journal of Molecular Neuroscience, 54(4), 796–802. 10.1007/s12031-014-0417-1 25267504

[brb31009-bib-0063] Nagata, T. , Shinagawa, S. , Nukariya, K. , Ochiai, Y. , Kawamura, S. , Agawa‐Ohta, M. , … Yamada, H. (2011). Association between brain‐derived neurotrophic factor (BDNF) gene polymorphisms and executive function in Japanese patients with Alzheimer’s disease. Psychogeriatrics, 11(3), 141–149. 10.1111/j.1479-8301.2011.00364.x 21951954

[brb31009-bib-0064] Nagata, T. , Shinagawa, S. , Nukariya, K. , Yamada, H. , & Nakayama, K. (2012). Association between BDNF polymorphism (Val66Met) and executive function in patients with amnestic mild cognitive impairment or mild Alzheimer disease. Dementia and Geriatric Cognitive Disorders, 33(4), 266–272. 10.1159/000339358 22699449

[brb31009-bib-0065] Narayanan, V. , Veeramuthu, V. , Ahmad‐Annuar, A. , Ramli, N. , Waran, V. , Chinna, K. , … Ganesan, D. (2016). Missense mutation of brain derived neurotrophic factor (BDNF) alters neurocognitive performance in patients with mild traumatic brain injury: A longitudinal study. PLoS ONE, 11(7), e0158838 10.1371/journal.pone.0158838 27438599 PMC4954696

[brb31009-bib-0066] Ng, T. , Teo, S. M. , Yeo, H. L. , Shwe, M. , Gan, Y. X. , Cheung, Y. T. , … Chan, A. (2016). Brain‐derived neurotrophic factor genetic polymorphism (rs6265) is protective against chemotherapy‐associated cognitive impairment in patients with early‐stage breast cancer. Neuro‐Oncology, 18(2), 244–251. 10.1093/neuonc/nov162 26289590 PMC4724179

[brb31009-bib-0067] Oroszi, G. , Lapteva, L. , Davis, E. , Yarboro, C. H. , Weickert, T. , Roebuck‐Spencer, T. , … Illei, G. G. (2006). The Met66 allele of the functional Val66Met polymorphism in the brain‐derived neurotrophic factor gene confers protection against neurocognitive dysfunction in systemic lupus erythematosus. Annals of the Rheumatic Diseases, 65(10), 1330–1335. 10.1136/ard.2006.051623 16606648 PMC1798324

[brb31009-bib-0068] Raz, N. , Rodrigue, K. M. , Kennedy, K. M. , & Land, S. (2009). Genetic and vascular modifiers of age‐sensitive cognitive skills: Effects of COMT, BDNF, ApoE, and hypertension. Neuropsychology, 23(1), 105–116. 10.1037/a0013487 19210038 PMC2729285

[brb31009-bib-0069] Richter‐Schmidinger, T. , Alexopoulos, P. , Horn, M. , Maus, S. , Reichel, M. , Rhein, C. , … Kornhuber, J. (2011). Influence of brain‐derived neurotrophic‐factor and apolipoprotein E genetic variants on hippocampal volume and memory performance in healthy young adults. Journal of Neural Transmission (Vienna, Austria: 1996), 118(2), 249–257. 10.1007/s00702-010-0539-8 21190051

[brb31009-bib-0070] da Rocha, F. F. , Malloy‐Diniz, L. , Lage, N. V. , & Correa, H. (2011). The relationship between the Met allele of the BDNF Val66Met polymorphism and impairments in decision making under ambiguity in patients with obsessive‐compulsive disorder. Genes, Brain, and Behavior, 10(5), 523–529. 10.1111/j.1601-183X.2011.00687.x 21401876

[brb31009-bib-0071] Rybakowski, J. , Borkoswka, A. , Czerski, P. , Skibinska, M. , & Hauser, J. (2003). Polymorphism of the brain‐derived neurotrophic factor gene and performance on a cognitive prefrontal test in bipolar patients. Bipolar Disorders, 5(6), 468–472. 10.1046/j.1399-5618.2003.00071.x 14636373

[brb31009-bib-0072] Rybakowski, J. , Borkoswka, A. , Skibinska, M. , Szczepankiewicz, A. , Kapelski, P. , & Leszcztnska, A. (2006). Prefrontal cognition in schizophrenia and bipolar illness in relation to Val66Met polymorphism of the brain‐derived neurotrophic factor gene. Psychiatry and Clinical Neurosciences, 60, 70–76. 10.1111/j.1440-1819.2006.01462.x 16472361

[brb31009-bib-0073] Schofield, P. R. , Williams, L. M. , Paul, R. H. , Gatt, J. M. , Brown, K. , Luty, A. , … Gordon, E. (2009). Disturbances in selective information processing associated with the BDNF Val66Met polymorphism: Evidence from cognition, the P300 and fronto‐hippocampal systems. Biological Psychology, 80(2), 176–188. 10.1016/j.biopsycho.2008.09.001 18838100

[brb31009-bib-0074] Strauss, K. , Barr, C. , & George, C. (2004). BDNF and COMT polymorphisms: Relation to memory phenotypes in young adults with childhood‐onset mood disorder. Neuromolecular Medicine, 5, 181–192. 10.1385/NMM:5:3:181 15626819

[brb31009-bib-0075] Stuart, K. , Summers, M. J. , Valenzuela, M. J. , & Vickers, J. C. (2014). BDNF and COMT polymorphisms have a limited association with episodic memory performance or engagement in complex cognitive activity in healthy older adults. Neurobiology of Learning and Memory, 110, 1–7. 10.1016/j.nlm.2014.01.013 24468545

[brb31009-bib-0076] Swardfager, W. , Herrmann, N. , Marzolini, S. , Saleem, M. , Shammi, P. , Oh, P. I. , … Lanctôt, K. L. (2011). Brain derived neurotrophic factor, cardiopulmonary fitness and cognition in patients with coronary artery disease. Brain, Behavior, and Immunity, 25(6), 1264–1271. 10.1016/j.bbi.2011.04.017 PMC4540594 21554945

[brb31009-bib-0077] Szabo, A. J. , Alosco, M. L. , Miller, L. A. , McGeary, J. E. , Poppas, A. , Cohen, R. A. , & Gunstad, J. (2013). Brain‐derived neurotrophic factor Val66Met polymorphism and cognitive function in persons with cardiovascular disease. Psychogeriatrics, 13(4), 206–212. 10.1111/psyg.12013 24289461 PMC3847660

[brb31009-bib-0078] Tan, Y. L. , Zhou, D. F. , Cao, L. Y. , Zou, Y. Z. , Wu, G. Y. , & Zhang, X. Y. (2005). Effect of the BDNF Val66Met genotype on episodic memory in schizophrenia. Schizophrenia Research, 77(2–3), 355–356. 10.1016/j.schres.2005.03.012 15913964

[brb31009-bib-0079] Thibeau, S. , McFall, G. P. , Wiebe, S. A. , Anstey, K. J. , & Dixon, R. A. (2016). Genetic factors moderate everyday physical activity effects on executive functions in aging: Evidence from the Victoria Longitudinal Study. Neuropsychology, 30(1), 6–17. 10.1037/neu0000217 26710092 PMC4693634

[brb31009-bib-0080] Thow, M. E. , Summers, M. J. , Summers, J. J. , Saunders, N. L. , & Vickers, J. C. (2017). Variations in the APOE allele or BDNF Val66Met polymorphism are not associated with changes in cognitive function following a tertiary education intervention in older adults: The Tasmanian Healthy Brain Project. Neurobiology of Aging, 55, 175–176. 10.1016/j.neurobiolaging.2017.03.028 28438485

[brb31009-bib-0081] Tukel, R. , Gurvit, H. , Ozata, B. , Oztürk, N. , Ertekin, B. A. , Ertekin, E. , … Direskeneli, G. S. (2012). Brain‐derived neurotrophic factor gene Val66Met polymorphism and cognitive function in obsessive‐compulsive disorder. American Journal of Medical Genetics. Part B, Neuropsychiatric Genetics: The Official Publication of the International Society of Psychiatric Genetics, 159B(7), 850–858. 10.1002/ajmg.b.32092 22911909

[brb31009-bib-0082] Uegaki, K. , Kumanogoh, H. , Mizui, T. , Hirokawa, T. , Ishikawa, Y. , & Kojima, M. (2017). BDNF binds its pro‐peptide with high affinity and the common Val66Met polymorphism attenuates the interaction. International Journal of Molecular Sciences, 18(5), pii: E1042 10.3390/ijms18051042 PMC5454954 28498321

[brb31009-bib-0083] Voineskos, A. , Lerch, J. , Felsky, D. , Shaikh, S. , Rajji, T. K. , Miranda, D. , … Kennedy, J. L. (2011). The brain derived neurotrophic factor Val66Met polymorphism and prediction of neural risk for Alzheimer disease. Archives of General Psychiatry, 68(2), 198–206. 10.1001/archgenpsychiatry.2010.194 21300947

[brb31009-bib-0084] Ward, D. D. , Summers, M. J. , Saunders, N. L. , Janssen, P. , Stuart, K. E. , & Vickers, J. C. (2014). APOE and BDNF Val66Met polymorphisms combine to influence episodic memory function in older adults. Behavioral Brain Research, 271, 309–315. 10.1016/j.bbr.2014.06.022 24946073

[brb31009-bib-0085] Ward, D. D. , Summers, M. J. , Saunders, N. L. , Ritchie, K. , Summers, J. J. , & Vickers, J. C. (2015). The BDNF Val66Met polymorphism moderates the relationship between cognitive reserve and executive function. Translational Psychiatry, 5, e590 10.1038/tp.2015.82 26125153 PMC4490292

[brb31009-bib-0086] Wegman, J. , Tyborowska, A. , Hoogman, M. , Arias Vásquez, A. , & Janzen, G. (2016). The BDNF Val66Met polymorphism affects encoding of object locations during active navigation. European Journal of Neuroscience, 45(12), 1501–1511. 27717213 10.1111/ejn.13416 PMC5484279

[brb31009-bib-0087] Wei, S. , Baller, E. , Kohn, P. , Kippenhan, J. , Klolachana, B. , Soldin, S. J. , … Berman, K. F. (2017). Brain‐derived neurotrophic factor Val66Met genotype and ovarian steroids interactively modulate working memory‐related hippocampal function in women: A multimodal neuroimaging study. Molecular Psychiatry, 23(4), 1066–1075. 28416813 10.1038/mp.2017.72 PMC10103851

[brb31009-bib-0088] Wei, S. M. , Eisenberg, D. P. , Kohn, P. D. , Kippenhan, J. S. , Kolachana, B. S. , Weinberger, D. R. , & Berman, K. F. (2012). Brain‐derived neurotrophic factor Val(6)(6)Met polymorphism affects resting regional cerebral blood flow and functional connectivity differentially in women versus men. Journal of Neuroscience, 32(20), 7074–7081. 10.1523/JNEUROSCI.5375-11.2012 22593075 PMC3362630

[brb31009-bib-0089] Wilkosc, M. , Szalkowska, A. , Sikibinska, M. , Zajac‐Lamparska, L. , Maciukiewciz, M. , & Araskiewicz, A. (2016). BDNF gene polymorphisms and haplotypes in relation to cognitive performance in Polish healthy subjects. Acta Neurobiologiae Experimentalis, 76, 43–52. 10.21307/ane-2017-004 27102917

[brb31009-bib-0090] Yang, B. , Ren, Q. , Zhang, J. C. , Chen, Q. X. , & Hashimoto, K. (2017). Altered expression of BDNF, BDNF pro‐peptide and their precursor proBDNF in brain and liver tissues from psychiatric disorders: Rethinking the brain‐liver axis. Translational Psychiatry, 7(5), e1128 10.1038/tp.2017.95 28509900 PMC5534963

[brb31009-bib-0091] Yin, Y. , Hou, Z. , Wang, X. , Sui, Y. , & Yuan, Y. (2015). The BDNF Val66Met polymorphism, resting‐state hippocampal functional connectivity and cognitive deficits in acute late‐onset depression. Journal of Affective Disorders, 183, 22–30. 10.1016/j.jad.2015.04.050 26000753

[brb31009-bib-0092] Yogeetha, B. S. , Haupt, L. M. , McKenzie, K. , Sutherland, H. G. , Okolicsyani, R. K. , Lea, R. A. , … Griffiths, L. R. (2013). BDNF and TNF‐alpha polymorphisms in memory. Molecular Biology Reports, 40(9), 5483–5490. 10.1007/s11033-013-2648-6 23918043

[brb31009-bib-0093] Yu, H. , Zhang, Z. , Shi, Y. , Bai, F. , Xie, C. , & Qian, Y. (2008). Association study of the decreased serum BDNF concentrations in amnestic mild cognitive impairment and the Val66Met polymorphism in Chinese Han. The Journal of Clinical Psychiatry, 69, 1104 10.4088/JCP.v69n0710 18505307

[brb31009-bib-0094] Zhang, X. , Chen, D. , Tan, Y. L. , Tan, S. , Luo, X. , Zuo, L. , & Soares, J. C. (2016). BDNF polymorphisms are associated with cognitive performance in schizophrenia patients versus healthy controls. Journal of Clinical Psychiatry, 7(8), 1011–1018. 10.4088/JCP.15m10269 27561148

[brb31009-bib-0095] Zhang, X. Y. , Chen, D. C. , Xiu, M. H. , Haile, C. N. , Luo, X. , Xu, K. , … Kosten, T. R. (2012). Cognitive and serum BDNF correlates of BDNF Val66Met gene polymorphism in patients with schizophrenia and normal controls. Human Genetics, 131(7), 1187–1195. 10.1007/s00439-012-1150-x 22362486 PMC3671849

